# Prevention Needs and Target Behavior Preferences in an App-Based Addiction Prevention Program for German Vocational School Students: Cluster Randomized Controlled Trial

**DOI:** 10.2196/59573

**Published:** 2025-06-24

**Authors:** Diana Guertler, Elaine Kraft, Dominic Bläsing, Anne Möhring, Christian Meyer, Hannah Schmidt, Florian Rehbein, Merten Neumann, Arne Dreißigacker, Anja Bischof, Gallus Bischof, Svenja Sürig, Lisa Hohls, Susanne Wurm, Stefan Borgwardt, Severin Haug, Hans-Jürgen Rumpf

**Affiliations:** 1 Department of Prevention Research and Social Medicine Institute for Community Medicine University Medicine Greifswald Greifswald Germany; 2 DZHK (German Centre for Cardiovascular Research), partner site Greifswald Greifswald Germany; 3 Department of Methods of Community Medicine Institute for Community Medicine University Medicine Greifswald Greifswald Germany; 4 Helmholtz Institute for One Health Greifswald Germany; 5 Department of Psychiatry and Psychotherapy University of Lübeck Lübeck Germany; 6 Department of Pediatrics University of Lübeck Lübeck Germany; 7 Department of Social Work FH Münster University of Applied Sciences Münster Germany; 8 Criminological Research Institute of Lower Saxony (KFN) Hannover Germany; 9 Swiss Research Institute for Public Health and Addiction University of Zürich Zürich Switzerland

**Keywords:** eHealth, computer tailoring, multiple addictive behaviors, vocational school students, module choice

## Abstract

**Background:**

Vocational school students exhibit a high prevalence of addictive behaviors. Mobile phone–based prevention programs targeting multiple addictive behaviors and promoting life skills are promising. Tailoring intervention content to participants’ preferences, such as allowing them to choose behavior modules, may increase engagement and efficacy. There is limited understanding of how personal characteristics relate to module choices.

**Objective:**

This study examined the prevention needs of German vocational school students as well as their prevention preferences through self-determined module choice in the multibehavior app-based addiction prevention program ready4life.

**Methods:**

A 2-arm cluster randomized controlled trial recruited German vocational school students aged ≥16 years. Among 376 classes from 35 schools, ready4life was introduced during a school lesson. Students were invited to download the ready4life app and completed an anonymous screening with individualized risk and competence feedback in the form of a traffic light system. Informed consent was provided by 2568 students. Intervention classes received individual app-based coaching with weekly chat contacts with a conversational agent over 4 months. They could choose 2 of 6 modules: alcohol, tobacco, cannabis, social media and gaming, stress, and social competencies. Control group classes received a link to health behavior information and could access coaching after 12 months.

**Results:**

Prevention need was high. For 86.2% (2213/2568), ≥2 risks were reported based on yellow or red traffic light feedback. Within the intervention group, stress (818/1236, 66.2%) and social media and gaming (625/1236, 50.6%) were the most chosen topics, followed by alcohol (360/1236, 29.1%), social competencies (306/1236, 24.8%), tobacco (232/1236, 18.8%), and cannabis (131/1236, 10.6%). Module choices closely aligned with received traffic light feedback among those with 1 or 2 risks. Multilevel regression models showed that women were significantly more likely to choose the stress module (odds ratio [OR] 2.38, 95% CI 1.69-3.33; *P*<.001); men preferred social media and gaming (OR 0.52, 95% CI 0.40-0.69; *P*<.001), alcohol (OR 0.50, 95% CI 0.37-0.67; *P*<.001), and cannabis (OR 0.37, 95% CI 0.21-0.63; *P*<.001) when holding age, educational track, and prevention need for the corresponding behavior constant. Younger students were significantly more likely to choose the cannabis module (OR 0.81, 95% CI 0.74-0.90; *P*<.001). Educational track also influenced module choice (eg, those with a lower educational level were more likely to choose alcohol and cannabis, suggesting a positive equity impact). Students’ prevention needs significantly influenced choice of the module (eg, higher alcohol consumption increased the likelihood of choosing the alcohol module; OR 1.31, 95% CI 1.20-1.43; *P*<.001).

**Conclusions:**

Our study confirms vocational school students’ high prevention needs regarding addictive behaviors. Students’ module choices were highly congruent to their demonstrated needs, with most students being interested in the stress module. Module choice also differed by age, gender, and educational track.

**Trial Registration:**

German Clinical Trials Register DRKS00022328; https://drks.de/search/en/trial/DRKS00022328

**International Registered Report Identifier (IRRID):**

RR2-10.1024/0939-5911/a000811

## Introduction

### Addictive Behaviors in Vocational School Students

Adolescence and young adulthood are periods of life associated with a number of challenges, including entering the world of work and increasing independence from parents. Addictive behaviors such as consumption of alcohol, tobacco, and cannabis [1] but also problematic internet use [2] are particularly widespread in this phase of life.

Many adolescents and young adults in Germany attend vocational schools to start an apprenticeship or attend vocational grammar school or vocational preparation courses. Vocational schools are promising settings for prevention efforts [3], especially as there is evidence that students in vocational schools report even higher rates of substance use than other adolescents and young adults. For example, in a survey of 5688 German vocational school students, the reported smoking rate was more than twice as high as that of their peers in the general population [4]. Representative data from German vocational school students in Mecklenburg-Western Pomerania also show particularly high rates of problematic alcohol consumption (62%), smoking (62%), and cannabis use (26%). The study [5] also provided a first insight into the prevalence of media-related problems among German vocational school students. Problematic internet use was reported by 17% of vocational school students, problematic gaming was reported by 10%, and potential smartphone addiction was reported by 17% [5]. This is supported by findings from an intervention trial to reduce problematic use of the internet in vocational school students [6].

In vocational school settings, achieving prevention goals typically requires a shift away from primary prevention strategies. Instead, indicated prevention programs are considered more appropriate as the vast majority of vocational school students have already initiated addictive behavior. A high proportion of vocational school students also engage in multiple health risk behaviors, especially multiple addictive behaviors [5,7]. However, they differ widely in their risk profiles (ie, in the combinations and number of addictive behaviors). Vocational school students also differ in other factors that may contribute to addictive behaviors, such as low social competence or high work stress. This means that complex interventions are needed to address different risk profiles.

### Tailored Mobile Interventions

Mobile interventions are best suited to address various risk profiles as they can be easily adapted to the needs of the users and allow for timely and anonymous participation. Mobile interventions have been shown to be effective in changing multiple addictive behaviors in vocational school students [8,9].

Mobile interventions have the advantage of automated delivery of highly individualized content through computer tailoring. Tailoring is a key component in ensuring the effectiveness of an intervention [10]. With tailoring, feedback that is relevant to the individual is derived from an individual assessment based on prespecified rules. Examples include content or wording tailored to users’ gender or motivation to change. It has been shown that tailored feedback compared to generic feedback is more likely to be processed and remembered [11]. In addition to automatic computer tailoring, some interventions include forms of self-tailoring, such as allowing participants to choose the medium [12], dose [13], or content (eg, specific behavioral targets or modules [14-17]). In line with theoretical frameworks such as self-determination theory, self-tailoring is assumed to increase autonomy [18].

Although mobile health interventions are effective, adherence is a major challenge [19], including approaches among vocational school students [20]. Low adherence may partly explain the lack of long-term effects and small effect sizes in mobile health interventions [21]. Allowing participants the autonomy to choose which behavioral modules to engage with could positively influence engagement and efficacy [22,23]. Within the vocational context, offering a choice of intervention modules could be valuable, particularly as many vocational school students exhibit multiple addictive behaviors [5,7]. Attempting to address all these behaviors simultaneously might be overwhelming [24], especially considering the decreasing intention to change all behaviors as their number increases [25].

### Mechanisms of Module Choice

While some research has explored participants’ module choices in multibehavior interventions [14,15,26-31], there is limited understanding of the underlying choice mechanisms, such as which personal characteristics are related to choice. Investigating this is crucial as diverse subgroups may use interventions differently and some may not use it as recommended (eg, not choosing the recommended modules). Such insights can inform tailored strategies to align interventions with participant needs, reducing nonadherence and enhancing intervention exposure and effectiveness.

Most of the evidence on module choice comes from multiple-behavior interventions in adults. These studies have shown that certain modules are preferred over others, for example, physical activity over diet [14,15,26]. There is also consistency that health-promoting behaviors such as physical activity and diet are preferred over substance-related modules [14,15,26-31]. In total, 2 studies among vocational school students reported on preferences within multiple–addictive behavior interventions. They showed that stress and non–substance-related behaviors such as social media use were chosen more often than substance-related behaviors such as smoking, alcohol or cannabis use [8,17].

With regard to potential determinants of module choice, the results are less consistent [14-16,27] and are likely to vary depending on the number and topics of available behavior modules to choose from, whether module recommendations were given, and whether any restrictions were placed on module choice (eg, how many modules could be chosen or whether modules that were not recommended could be chosen). Providing feedback or recommending modules may enhance the alignment between participants’ module choice and their prevention needs [30]. Studies show that people are more inclined to choose modules that were recommended to them based on their demonstrated risk profile [17,29]. Consequently, most previous studies have used feedback or recommendations to guide module choice [14-17,26,29,32], with only a few exceptions [27,28,30,33].

In online multiple-behavior interventions for adults addressing a range of health-promoting behaviors and substance use, choice of the physical activity– or diet-related modules was higher among women [14] and those with healthier lifestyles [15]. In the study by Schulz et al [15], choice of the alcohol module was linked to being a man, lower income, and higher psychosocial distress, whereas medium (vs high) educational level, lower quality of life, and lower psychosocial distress were linked to choice of the smoking module. Reinwand et al [34] showed that adherence to module recommendations in online multibehavior interventions (ie, starting all recommended modules vs fewer) also depended on individual characteristics. Individuals were more likely to start all the recommended modules if they were older, a woman, unemployed, ill, and in a relationship.

The main gap in the existing research is that no study has reported on the determinants of module preferences among vocational school students or statistically analyzed the alignment between prevention needs and module preferences in this group. This is particularly important given that a high proportion of vocational school students engage in multiple addictive behaviors. Furthermore, although there have been previous studies on the prevalence of addictive behaviors among German vocational school students [5,35], these studies did not analyze prevention needs in terms of life skills and their determinants. This has only been investigated in a Swiss sample [17].

Given the fact that lower educational attainment among vocational school students is associated with higher prevalence [35] and clustering [5] of addictive behaviors, as well as lower engagement with mobile interventions [36], it is particularly important to analyze educational track as a potential determinant of module choice.

### Aims

This study examined the prevention needs of German vocational school students as well as their module choices within the app-based addiction prevention intervention ready4life. The ready4life intervention offers 6 behavior modules: alcohol, tobacco, cannabis, social media and gaming, stress, and social competencies. After receiving feedback on their individual risks and competencies in the form of a traffic light system to guide choice, students could freely choose 2 out of the 6 modules to be coached in. In terms of module choice, we aimed to examine (1) how often each intervention module was chosen, (2) how module choice aligned with the traffic light feedback received, and (3) whether individual characteristics (age, gender, educational track, and prevention needs) were associated with module choice. In addition, the relationship between sociodemographic factors and module choice is likely to be influenced by the prevention needs of the students in this sample. Therefore, we also analyzed how sociodemographics (age, gender, and educational track) were associated with prevention needs. This would provide a clearer understanding of how these factors may shape students’ intervention preferences.

The prevention needs of vocational school students in terms of addictive behaviors were expected to be high [5]. On the basis of previous literature on module choice [8,14,15,17,26-31] and the generally low intentions to change addictive behaviors [5], it was expected that life skill modules (stress or social competencies) would be preferred over modules related to addictive behaviors. It was also expected that module choice would show a high alignment with the traffic light feedback received [8,17,29]. Given the differences in prevalence of substance use between the genders [37], it was expected that men would choose modules related to substance use more often than women.

## Methods

### Design

The data came from a cluster randomized controlled trial among German vocational school students testing the efficacy of the app-based addiction prevention program ready4life [38]. The trial was registered in the German Clinical Trials Register (DRKS00022328).

### Participants and Procedures

#### Recruitment

Details on recruitment can be found elsewhere [39]. Participants in this study were vocational school students from 5 German federal states who were enrolled in vocational training, vocational preparation, or vocational grammar school. Randomization was conducted at the class level within computer-generated blocks of 4 and stratified by school (allocation ratio: 1:1). Within the 35 participating schools, 376 classes were randomized to either the intervention (n=186, 49.5%) or control (n=190, 50.5%) condition.

We used a facilitated access approach by proactively offering intervention participation during school hours. Combining mobile interventions with proactive contacting is crucial to maximize the reach and representativeness of the sample [40], particularly for those not yet planning to change their behavior [41]. This is of high importance as most vocational school students do not yet intend to change their addictive behaviors [5].

#### Class Introductions

During 1 to 2 school hours, students from both study groups were introduced to the study and were invited to download the ready4life app. During the introduction, students were given detailed information about the study, including its procedures, randomization process, data protection measures, and the content and features of the app. To minimize stigma associated with addiction, introductions largely focused on stress management. Class introductions were conducted for a total of 376 classes between October 2020 and March 2022. Of these, 63.6% (239/376) of classes were introduced by members of the project team, 14.9% (56/376) by trained schoolteachers, 9% (34/376) by addiction prevention experts, 5.9% (22/376) by school social workers, and 6.6% (25/376) by vocational school students. The introductions were delivered via face-to-face (187/376, 49.7%), online streaming (162/376, 43.1%), or an emailed YouTube video link (27/376, 7.2%).

#### Study Participation

Vocational school students aged ≥16 years with smartphones and who provided contact details (email or phone) for follow-up data collection were eligible for study participation. After downloading the app, all students participated in an anonymous in-app screening on their prevention needs regarding alcohol, tobacco, and cannabis consumption; internet use; social competencies; and stress. Students then provided digital informed consent. A total of 4225 app downloads were recorded, and 2568 students provided informed consent. Participation rate was 46.7% (2508/5370) among students aged ≥16 years who had the correct app version, and belonged to classes where the number of students present during the introduction was known.

All study participants received feedback on their individual risks and competencies in the form of a traffic light system and were then informed of their group allocation. The intervention group then received app-based coaching for 2 self-selected behavior modules over 16 weeks (see the Interventions section for details).

#### Follow-Up Assessment

Students from both study groups were invited to 2 follow-up sessions at 6 and 12 months via SMS text message or email with a link to an online chat conversation with a virtual coach. If students did not respond after reminders, they were contacted by the project team to provide the option to complete the follow-up during a telephone interview. Prizes were raffled after the 12-month follow-up to increase participant engagement. Prizes were drawn in cash instead of vouchers due to procurement constraints. Participants in both the intervention and control groups with available contact information were eligible. The raffle was based on credits earned on the app, with separate raffles for each group to avoid disadvantaging the control group. Participants were randomly selected for the prizes, which ranged from €10 to €500 (US $11.33 to US $566.39).

### Instruments

#### Class Registration Data

Class-level information was gathered during the registration process for classes, encompassing the federal state of the school, the classes’ educational track (vocational preparation, vocational training, and vocational grammar school), year of education, class size, and data concerning the introduction (eg, number of students present, mode of introduction, or introducing person). For students undergoing vocational training, the official label of their training was assessed, and occupations were classified using the German Classification of Occupations [42] (Table S1 in Multimedia Appendix 1). A recode table was used to convert these into the International Standard Classification of Occupations 2008 (ISCO-8) [43], which was used in the main analysis to ensure international compatibility (Table S2 in Multimedia Appendix 1).

Vocational preparation classes are designed for students with basic education to qualify for further vocational training. Vocational grammar schools, on the other hand, allow students to obtain a university entrance qualification. Vocational training typically combines part-time classroom study with on-the-job experience. Entry requirements vary, leading to differences in the socioeconomic backgrounds of students on different tracks. Tracks leading to operational and support roles (ie, clerical support workers, service and sales workers, craft-related trade workers, plant and machine operators and assemblers, and elementary occupations) generally require lower academic qualifications and attract students of a lower socioeconomic status. For example, individuals from lower-income backgrounds often choose these tracks because of financial constraints, limited access to higher education, or expectations of early entry into the labor market. The lower socioeconomic status of students in these tracks also partly results from the lower prestige and job security associated with these tracks, compared to tracks leading to professional, technical, and associate professional roles.

#### Individual Screening Data

Within the app-based screening, data at the individual level were collected, including sociodemographics, addictive behaviors (alcohol, tobacco, and cannabis consumption and problematic internet use), perceived stress, and social competencies.

#### Sociodemographics

Age was calculated from the date of birth given by the students. Gender was assessed as man, woman, or other. When students selected “other,” we assessed their tendency to feel more masculine or feminine on a scale from 1 to 6. Due to the limited number of students in the category of “other” (40/2568, 1.6%), those who tended to be more masculine (scores of 1-3; 17/40, 42%) were categorized as men, and those who tended to be more feminine (scores of 4-6; 23/40, 58%) were categorized as women.

#### Alcohol Consumption

Using questions from the Alcohol Use Disorders Identification Test–Consumption scale [44], students reported their frequency of alcohol consumption in the previous 30 days (0-30). For those who reported consumption, details of the type of drink, the number of drinks consumed on a typical day, and the day with the most alcoholic drinks consumed in the last month were collected using a digital bar displaying common portion sizes (eg, 0.5-L beer or 2-cL shot). Standard drinks were calculated from the selected drinks (1 standard drink=12 g of alcohol). A quantity-frequency index was calculated by multiplying drinking days by typical standard drinks per day and dividing by 30.

#### Tobacco Consumption

Frequency of smoking tobacco or using nicotine products in the previous 30 days was assessed, with response options including “(almost) daily,” “occasionally but not daily,” or “never.” For those who reported smoking, the number of consumption days (0-30) and cigarettes smoked per day in the previous month was assessed. A quantity-frequency index was calculated by multiplying days of use by cigarettes smoked per day and dividing by 30.

#### Cannabis Consumption

Lifetime use of cannabis containing tetrahydrocannabinol was assessed using the response options “no, never” and “yes.” For those who reported cannabis use, frequency in the previous 6 months was assessed (“not at all,” “once a month or less,” “2-4 times a month,” “2-3 times a week,” and “4 times a week or more”), as well as the number of days of use in the previous month (0-30).

#### Problematic Internet Use

The short version of the Compulsive Internet Use Scale (CIUS) [45] measured problematic internet use on a scale from 0 (“never”) to 4 (“very often”). A total score was calculated with a possible range of 0 to 20. Sum scores of ≥7 indicate a problematic use of the internet.

#### Stress

Stress was assessed using a single-item questionnaire [46]: “Stress is a state in which a person feels tense, restless, nervous, or anxious, or is unable to sleep at night due to disturbing thoughts. How much do you currently feel this type of stress?” Response options ranged from 1 (“not at all strong”) to 5 (“very strong”).

#### Social Competencies

On the basis of the Assertion Inventory [47], 8 items measured social competence in approaching others, expressing needs, resisting group pressure, and standing up for oneself. Response options ranged from 1 (“very uncertain”) to 5 (“very certain”). A sum score ranging from 8 to 40 was calculated, with higher values indicating higher social competence.

#### Individual Risk and Competence Feedback and Module Choice

The received traffic light feedback (green, yellow, and red) for each behavior and the 2 chosen behavior modules were automatically recorded on the app for each student. For each module, 1 dichotomous variable was created indicating whether it was chosen (see the Interventions section for details).

### Interventions

#### Development

Details on intervention development have been published elsewhere [38]. The coaching app ready4life was initially developed and evaluated in Switzerland aiming to prevent or reduce addictive behaviors and promote life skills among vocational school students [48]. The latest version of ready4life was further enhanced for this study. In particular, in addition to the 4 existing modules on alcohol, tobacco, stress management, and social competencies, 2 new modules focusing on cannabis and social media and gaming were developed [38]. The adapted version of the ready4life app used in this study consisted of the app-based screening followed by the individual risk and competence feedback and the app-based coaching.

#### Efficacy

We have shown that the adapted version of ready4life is effective in reducing problematic internet use, stress, and tobacco use, as well as in improving social competencies over 12 months in this study’s sample, which will be published elsewhere. Similarly, among Swiss vocational school students, the 6-module ready4life version significantly reduced at-risk drinking and problematic internet use over 6 months [9].

#### Individual Risk and Competence Feedback

**Figure 1:**
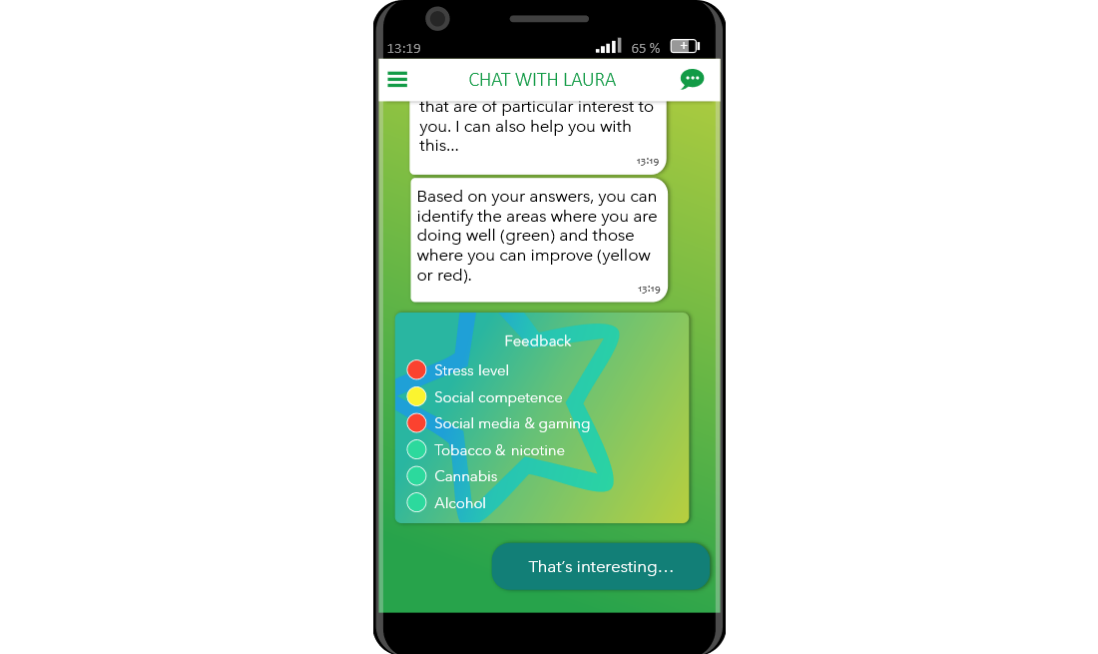
Individual risk and competence feedback provided by the app (English translation).

All study participants received in-app feedback on their individual risks and competencies in the form of a traffic light system for each assessed behavior of the in-app screening (Figure 1). Students received a green traffic light when they reported no or minor risks (eg, did not smoke tobacco or use nicotine products in the previous 30 days or perceived no or low stress) or a high competence (eg, feeling secure in social situations) concerning the specific behavior. A yellow traffic light was given if students reported a moderate risk or competence (eg, occasional tobacco smoking or nicotine product use in the previous 30 days, medium perceived stress, and feeling insecure in social situations), and a red traffic light was given if students reported a high risk or low competence (eg, daily or almost daily tobacco smoking or nicotine product use in the previous 30 days, high perceived stress, and feeling highly insecure in social situations). For social media and gaming, students received yellow traffic light feedback if they had a Short CIUS score of ≥7 [45], indicating a problematic internet use. The red traffic light used a cutoff of ≥9, which has been shown to have a higher specificity. Feedback on alcohol use was based on age- and gender-specific cutoffs derived from established guidelines on alcohol consumption [49]. Topic-specific thresholds for receiving a green, yellow, or red traffic light are shown in Table S3 in Multimedia Appendix 1.

#### Module Choice and App-Based Coaching

Students from the intervention group chose 2 out of 6 available behavior modules (alcohol, tobacco, cannabis, social media and gaming, stress, and social competencies) to be coached in for 16 weeks via the ready4life app as described in this section. Because many vocational school students engage in multiple addictive behaviors, addressing all at-risk behaviors could be overwhelming [24]. Therefore, the choice of modules was limited to 2. Previous research suggests that this may optimize adherence to module recommendations [34] and improve intervention efficacy [24]. Given the comprehensive approach of the intervention, we expected that focusing on 2 selected modules would positively influence unselected topics by increasing students’ motivation and self-efficacy to subsequently address other behaviors in the long term. In addition, we anticipated that changes in one behavior would positively influence other behaviors (eg, by improving resilience factors such as social competence and stress management, which are known to protect against addiction [50,51]).

The students were advised to use their individual risk and competence feedback to guide their module choice (eg, choosing modules on behaviors for which they had received yellow or red traffic light feedback). However, module choice was completely self-determined, including the option to choose modules on behaviors for which students had received green traffic light feedback.

Each module included 8 weeks of coaching. The 2 selected modules could not be started simultaneously. Instead, we chose to present the modules sequentially for the following reasons: (1) gradual introduction of behaviors may reduce participants’ burden and resistance, thereby improving engagement [23] and efficacy [52]; (2) success in changing one behavior may increase students’ confidence in tackling others subsequently; (3) this approach is consistent with goal setting theory by breaking goals down into manageable steps [53]; and (4) while simultaneous approaches may work well with populations with a high willingness to change multiple behaviors, a sequential approach may be particularly beneficial for populations with lower motivation, such as vocational school students [5]. The 2 chosen modules were presented in a random order.

The app-based coaching involved weekly chat interactions (once a week for approximately 5 min) over a 4-month period. A rule-based conversational agent (ie, without artificial intelligence) provided individualized feedback within the selected intervention modules. Chats also included media (videos, images, and links), quizzes, and contests. The content of the chats was constructed using the theoretical frameworks of social cognitive theory [54] and the health action process approach [55]. Each weekly session addressed specific behavioral constructs and goals, such as self-monitoring, risk perception, goal setting, or coping strategies. The detailed weekly content can be found in the study by Schmidt et al [38].

Predefined decision rules guided the chat interactions, with participant input during the conversations determining the subsequent responses of the conversational agent. For example, participants actively chose whether to receive additional information on certain topics, and the agent’s responses varied depending on the accuracy of the participant’s answers to quiz questions. Feedback was further tailored using participant data collected during the app-based screening. These data included demographic variables (eg, age and gender), behavioral indicators (eg, level of alcohol consumption and social competencies), and other variables such as motivation to change the targeted behavior or perceived pros and cons of behavior change. In addition, progress in behavior change was assessed at weeks 8 and 16 to allow for ipsative feedback.

In addition to the weekly chats, users could self-initiate chat dialogues at any time. Push notifications (eg, “Hi <nickname>”) were used to inform the students of new chat content once a week. Further reminders were used for contest participation or if the app was not used for an extended period. Personal support in the form of an “ask-the-expert” function was integrated into the app.

#### Control Group

After participating in the in-app screening and receiving the individual risk and competence feedback, students from the control group only received a link to information on enhancing health behaviors. However, they could access module choice and app-based coaching after 12 months.

Reporting checklists can be found in Multimedia Appendices 2-4.

### Data Analyses

#### Description of the Sample and Prevention Needs

Descriptive statistics were used to describe the sample using numbers and percentages, means and SDs, or medians and IQRs for highly skewed data. Prevention needs were described in terms of continuous addictive behaviors, stress, and social competencies. Age, gender, and educational track were analyzed as potential determinants of students’ prevention needs using multiple multilevel regression models.

#### Individual Risk and Competence Feedback

The percentages of students receiving green, yellow, or red traffic light feedback were graphically displayed for each behavior. The percentages of students showing different numbers of risks were reported based on yellow or red traffic light feedback and red traffic light feedback only.

#### Module Choice

Of the 1286 participants randomized to the intervention group, 20 (1.6%) had no access to the intervention due to mistyping of their class password, and furthermore, 30 (2.3%) did not choose modules. Thus, module choice was analyzed for 96.1% (1236/1286) of the intervention group participants.

The percentages of participants who chose each behavior module were reported for all intervention participants, intervention participants who received yellow or red traffic light feedback for that behavior, and intervention participants who received red traffic light feedback. As only 2 out of 6 behavior modules could be chosen, percentages of intervention participants who chose each behavior module were also reported based on the number of shown risks (1 or 2 risks or >2 risks).

Congruence between presented risk and module choice was measured using the proportion of students with yellow or red traffic light feedback for a particular behavior (eg, alcohol) among those who selected the corresponding behavior module (eg, alcohol module).

#### Potential Determinants of Module Choice

Multiple multilevel logistic regression analyses were conducted to model choice of each module separately (1=module chosen; 0=not chosen). Potential determinants included age, gender, educational track, and the corresponding prevention need (ie, problematic internet use was included as the independent variable for modeling choice of the social media and gaming module, social competencies were included as the independent variable when modeling choice of the social competencies module, and so on). All regression models included random intercepts at the class level [56] to account for the clustered structure of the data. Intraclass correlation coefficients (ICCs) were computed using an intercept-only model. ICCs express the percentage of the total variance in the outcome (eg, module choice) that is attributed to class membership [56]. Partly low numbers of classes per school prohibited modeling of random intercepts at the school level. Significance was set at *P*<.05. All statistical analyses were conducted using Stata SE (version 17.0; StataCorp).

#### Use of the German Classification of Occupations to Analyze Determinants of Prevention Needs and Module Choice

A sensitivity analysis was conducted using the German Classification of Occupations [42]. The German Classification of Occupations differs from the ISCO-8 in that it classifies occupations based on the specific content, industry, or sector in which the work is performed compared to the ISCO-8, which categorizes occupations based on the tasks, skills, and responsibilities associated with them. It considers the German labor market’s unique characteristics and is more detailed in classifying occupations within the context of Germany.

### Ethical Considerations

Ethics approval was granted by the ethics committees of the University of Lübeck (19-419) and the University Medicine Greifswald (BB 024/20). Digital informed consent was obtained from vocational school students through the app, with no additional parental consent necessary in accordance with the European Union General Data Protection Regulation. Underaged participants were encouraged to inform their parents or guardians about study participation. Students completed the app-based screening anonymously. No names were collected; instead, students chose nicknames. Data protection was ensured through encrypted data transmission and storage within the app. For those who provided informed consent, research data were stored in a pseudonymized format, and contact information (email or phone number) was securely stored on a separate server. Participants were not offered any compensation other than the chance to win prizes.

## Results

### Sample Description and Prevention Needs

**Figure 2:**
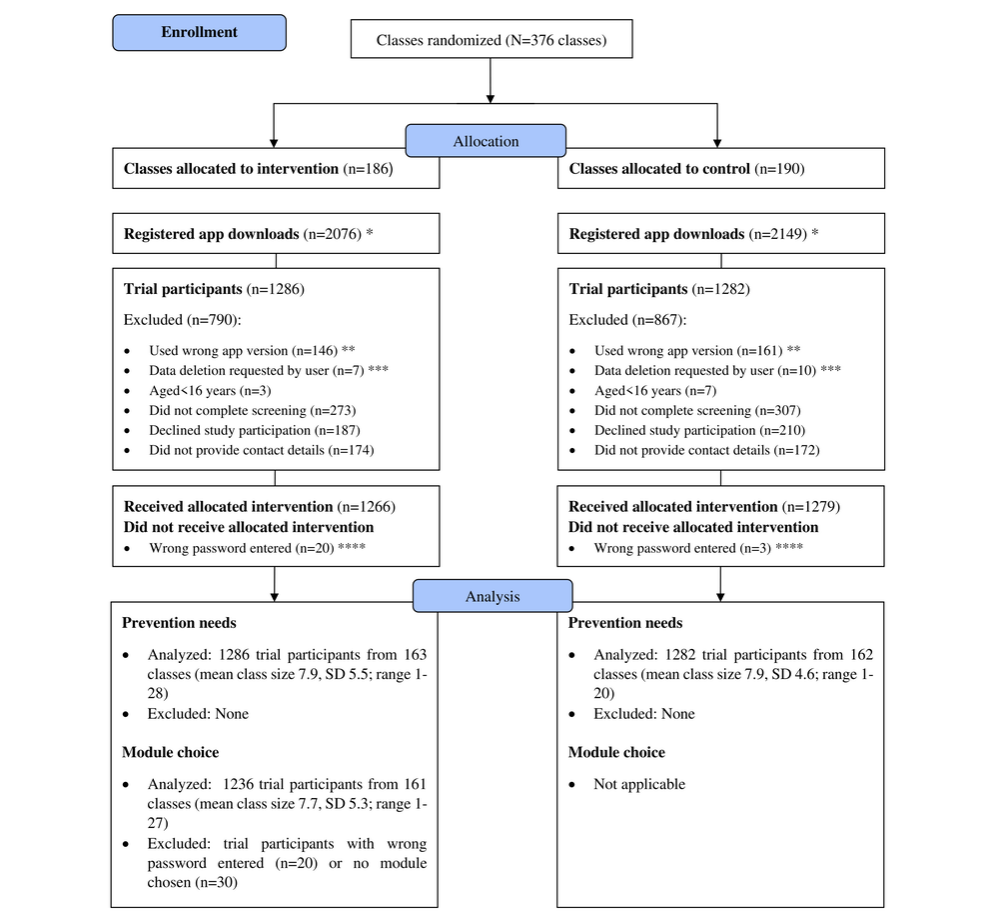
Flowchart of clusters and participants. *n=11 students were identified who had registered for the study twice; these were already subtracted from the download count. **The app included multiple versions tailored to different countries or languages. Due to a technical issue, between September 2, 2021, and December 10, 2021, a total of 307 students were mistakenly directed to a different version of the app instead of the German version. As a result, these students used a version of the app that was not part of this study, and informed consent could not be obtained from them. ***App users were able to initiate the deletion of all data collected at any time point regardless of whether they had just started the screening or provided informed consent and were using the intervention already. ****Group allocation was conducted at the class level based on class-specific passwords; due to mistyping of passwords, 1.6% (20/1286) of the students from the intervention group did not receive access to the intervention, and 0.2% (3/1282) of the students from the control group received access to the intervention.

The flow of the participants is depicted in Figure 2. Study participants are described in Table S4 in Multimedia Appendix 1. Among the total sample, the mean age was 19.7 (SD 3.7) years, and 45.6% (1170/2568) were women. Most study participants (1648/2568, 64.2%) were in vocational training, followed by vocational grammar school (582/2568, 22.7%) and vocational preparation (282/2568, 11%). Within those students in vocational training, the most prevalent occupations were technicians and associate professionals (511/1648, 31%), as well as craft-rated trade workers (422/1648, 25.6%). Most students (1785/2568, 69.5%) were in their first or second year of education.

In terms of prevention needs, students on average perceived a moderate level of stress (mean 3.29, SD 1.20 on a scale from 1 to 5) and reported a moderate level of social competencies (mean 29.50, SD 4.69 on a scale from 8 to 40). The mean score on the Short CIUS was 9.14 (SD 4.18), which is above the threshold for problematic use of the internet. The median number of standard alcoholic drinks consumed per day was 0.20 (IQR 0.00-1.00). A proportion of 31.7% (814/2568) and 18.1% (464/2568) had used tobacco and cannabis in the previous month, respectively.

Of the total sample, 45.1% (1158/2568) of the students used the app on Android, and 54.9% (1410/2568) used it on iOS. Notably, iOS users reported significantly higher prevention needs across all behavioral domains except social competencies, where they had lower needs compared to Android users. In addition, iOS users were more likely to select the tobacco module (data not shown).

When analyzing prevention needs as outcomes, ICCs were highest for alcohol consumption, indicating that 13.5% of the variance in the number of standard alcoholic drinks per day was attributable to class membership (Table S5 in Multimedia Appendix 1). Being a woman was associated with significantly higher stress (regression coefficient=0.70, 95% CI 0.60-0.80; *P*<.001), higher problematic internet use (regression coefficient=0.74, 95% CI 0.39-1.09; *P*<.001), and lower social competencies (regression coefficient=−2.27, 95% CI −2.67 to −1.88; *P*<.001). In contrast, being a woman was also associated with lower substance use (lower alcohol consumption: regression coefficient=−0.40, 95% CI −0.55 to −0.24 and *P*<.001; lower odds of tobacco use: odds ratio [OR] 0.79, 95% CI 0.65-0.97 and *P*=.02; lower odds of cannabis use: OR 0.60, 95% CI 0.48-0.75 and *P*<.001) holding age and educational track constant.

Higher problematic internet use was significantly associated with younger age (regression coefficient=−0.10, 95% CI −0.15 to −0.06; *P*<.001), whereas higher odds of tobacco use were associated with older age (OR 1.04, 95% CI 1.01-1.06; *P*=.006). Stress (regression coefficient=0.01, 95% CI −0.001 to 0.03; *P*=.06), social competencies (regression coefficient=0.05, 95% CI −0.005 to 0.10; *P*=.07), odds of cannabis use (OR 1.02, 95% CI 0.99-1.05; *P*=.18), and alcohol consumption (regression coefficient=0.01, 95% CI −0.004 to 0.03; *P*=.12) were not significantly associated with age.

Wald tests indicated that social competencies (*P*=.02), problematic internet use (*P*=.002), and the odds of tobacco use (*P*<.001) were significantly associated with educational track after adjusting for gender and age, whereas perceived stress (*P*=.26), odds of cannabis use (*P*=.68), and alcohol consumption (*P*=.54) did not differ significantly between educational tracks. Clerical support workers (regression coefficient=1.10, 95% CI 0.36-1.84; *P*=.004), service and sales workers (regression coefficient=0.79, 95% CI 0.05-1.52; *P*=.04), students in vocational grammar school (regression coefficient=0.89, 95% CI 0.27-1.52; *P*=.005), and students in vocational preparation (regression coefficient=1.02, 95% CI 0.26-1.79; *P*=.009) reported significantly higher social competencies than the reference group of professionals, technicians, and associate professionals. The other educational tracks did not differ significantly in their social competencies from each other (data not shown).

Problematic internet use was significantly lower among craft-related trade workers (regression coefficient=−0.76, 95% CI −1.35 to −0.17; *P*=.01), and the odds of tobacco use were significantly higher among service and sales workers (OR 1.61, 95% CI 1.10-2.37; *P*=.02) than in the reference group.

Comparisons between the other educational tracks revealed that service and sales workers (regression coefficient=−0.81, 95% CI −1.57 to −0.06; *P*=.03), as well as craft-related trade workers, plant and machine operators and assemblers, and elementary occupations (regression coefficient=−1.26, 95% CI −1.93 to −0.58; *P*<.001), reported significantly lower problematic internet use than clerical support workers, whereas students in vocational grammar school (regression coefficient=0.95, 95% CI 0.38-1.52; *P*=.001) and vocational preparation (regression coefficient=1.00, 95% CI 0.30-1.69; *P*=.005) reported significantly higher problematic internet use than craft-related trade workers, plant and machine operators and assemblers, and elementary occupations. Vocational grammar school students also had lower odds of tobacco use than service and sales workers (OR 0.46, 95% CI 0.31-0.68; *P*<.001); craft-related trade workers, plant and machine operators and assemblers, and elementary occupations (OR 0.52, 95% CI 0.37-0.74; *P*<.001); and students in vocational preparation (OR 0.64, 95% CI 0.43-0.95; *P*=.03). Finally, service and sales workers had higher odds of tobacco use than clerical support workers (OR 1.61, 95% CI 1.03-2.49; *P*=.04).

### Individual Risk and Competence Feedback

**Figure 3:**
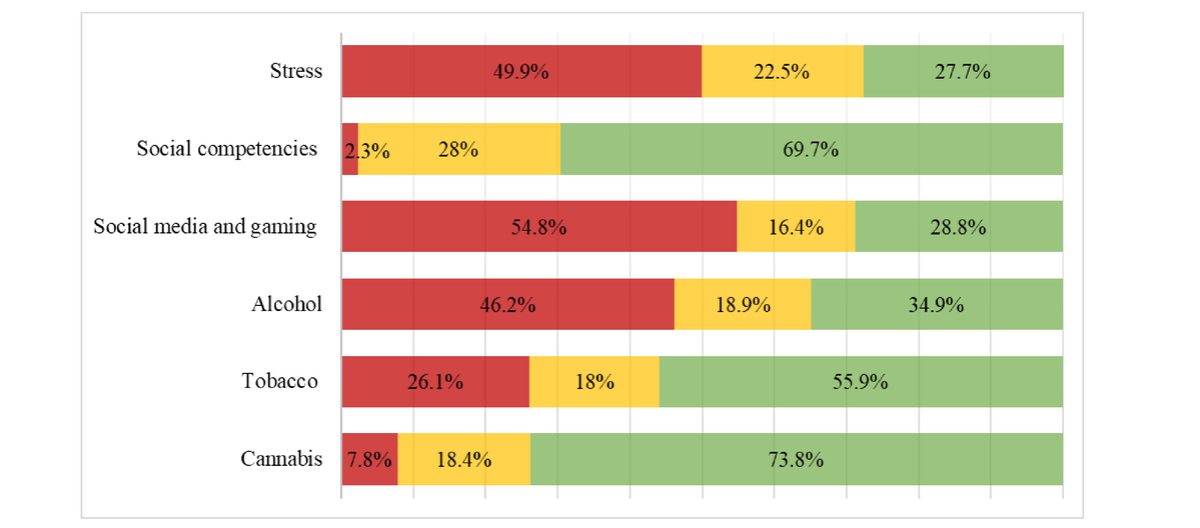
Percentage of students who received red, yellow, or green traffic light feedback by topic (N=2568). Traffic light feedback was based on self-reported behaviors and gave advice on potentially useful modules.

Figure 3 shows the percentage of study participants who received red, yellow, or green traffic light feedback for each behavior. Approximately two-thirds of the study participants received either red or yellow traffic light feedback for stress (1858/2568, 72.4%), social media and gaming (1828/2568, 71.2%), or alcohol (1672/2568, 65.1%). For tobacco, less than half (1133/2568, 44.1%) of the study participants received red or yellow traffic light feedback, whereas for cannabis (673/2568, 26.2%) and social competencies (778/2568, 30.3%), only approximately one-third received red or yellow traffic light feedback.

On average, students received red or yellow traffic light feedback for 3.09 (SD 1.42) of the 6 behaviors and red traffic light feedback only for 1.87 (SD 1.17) of the 6 behaviors. Only 2.7% (70/2568) of the study participants received neither red nor yellow traffic light feedback, and 11.1% (286/2568) received no red traffic light feedback for any of the 6 behaviors. The number of participants receiving red or yellow traffic light feedback for 1, 2, 3, 4, 5, and 6 behaviors were 11.1% (285/2568), 21.42% (550/2568), 24.8% (637/2568), 22.6% (580/2568), 13.6% (350/2568), and 3.7% (96/2568), respectively. The number of participants receiving red traffic light feedback for 1, 2, 3, 4, 5, and 6 behaviors were 30% (769/2568), 30.5% (783/2568), 19% (488/2568), 7.9% (204/2568), 1.4% (37/2568), and 0.04% (1/2568), respectively.

### Module Choice

Among students who made a module choice, the most frequently chosen coaching topic was stress (818/1236, 66.2%), followed by social media and gaming (625/1236, 50.6%), alcohol (360/1236, 29.1%), social competencies (306/1236, 24.8%), tobacco (232/1236, 18.8%), and cannabis (131/1236, 10.6%). The frequency of module choice was higher among those students who had received red or yellow traffic light feedback for the corresponding behavior than among the total group (Table 1). This trend was highest for stress, with 80.3% (727/905) of students with yellow or red traffic light feedback and 87.7% (541/617) of students with red traffic light feedback only choosing the stress module. The lowest choice rate was found for alcohol consumption. Even within those with red traffic light feedback, only 46.6% (274/588) chose the alcohol module.

**Table 1:** Frequency of participants’ module choice by traffic light feedback. Traffic light feedback was based on self-reported behaviors and gave advice on potentially useful modules; however, vocational school students could freely select any 2 of the 6 modules (N=1236).

Module	Any feedback (n=1236), n (%)	Yellow or red traffic light feedback, n/N (%)	Red traffic light feedback, n/N (%)
Stress	818 (66.2)	727/905 (80.3)	541/617 (87.7)
Social competencies	306 (24.8)	188/368 (51.1)	22/27 (81.5)
Social media and gaming	625 (50.6)	541/882 (61.3)	451/687 (65.7)
Alcohol	360 (29.1)	344/820 (42)	274/588 (46.6)
Tobacco	232 (18.8)	226/552 (40.9)	189/327 (57.8)
Cannabis	131 (10.6)	117/323 (36.2)	68/94 (72.3)

Table S6 in Multimedia Appendix 1 shows that, among those students with only 1 or 2 instances of yellow or red traffic light feedback, the choice rates for the recommended modules ranged from 68% (13/19) to 98% (40/41). For those with >2 instances of yellow or red traffic light feedback, the choice rates were much lower as students had to choose between several recommended modules. For example, choice rate for social media and gaming among those with >2 instances of yellow or red traffic light feedback (193/206, 93.7%) was approximately 30% lower than among those with 1 or 2 instances of yellow or red traffic light feedback (542/884, 61.3%). However, the number for some of the subsamples was rather small.

Most students chose modules that were congruent with their presented risks. For example, 88.9% (727/818) of the students who chose the stress module had yellow or red traffic light feedback in this behavior area. The congruence rates for the other behaviors were 61.4% (188/306) for social competencies, 86.6% (541/625) for social media and gaming, 95.6% (344/360) for alcohol, 97.4% (226/232) for tobacco, and 89.3% (117/131) for cannabis.

### Potential Determinants of Module Choice

Table S7 in Multimedia Appendix 1 shows the association between gender, age, educational track, and prevention needs and module choice. The ICCs for the modules ranged from 2.1% to 11%, indicating a modest influence of class membership on module choice.

Women were significantly more likely to select the stress module than men (OR 2.38, 95% CI 1.69-3.33; *P*<.001), who were more likely to select social media and gaming (OR 0.52, 95% CI 0.40-0.69; *P*<.001), alcohol (OR 0.50, 95% CI 0.37-0.67; *P*<.001), and cannabis (OR 0.37, 95% CI 0.21-0.63; *P*<.001) than women when holding age, educational track, and prevention need for the corresponding behavior constant. Men and women did not differ significantly with respect to their frequency of module choice for the tobacco (OR 1.01, 95% CI 0.70-1.46; *P*=.97) and social competencies (OR 1.13, 95% CI 0.83-1.54; *P*=.44) modules. Younger students were significantly more likely to choose the cannabis module (OR 0.81, 95% CI 0.74-0.90; *P*<.001), whereas there were no significant differences in module choice by age for the other 5 modules.

Although Wald tests did not reach significance, there were individual educational tracks that differed in their odds of choosing the social competencies, social media and gaming, alcohol, and cannabis modules when compared to the reference category. Craft-related trade workers, plant and machine operators and assemblers, and elementary occupations had a significantly lower likelihood of choosing social media and gaming when compared to the reference group of professionals, technicians, and associate professionals (OR 0.61, 95% CI 0.39-0.97; *P*=.04), whereas the other tracks did not differ significantly from the reference group in their likelihood of choosing social media and gaming. Clerical support workers (OR 1.85, 95% CI 1.02-3.38; *P*=.04); service and sales workers (OR 1.77, 95% CI 1.00-3.15; *P*=.049); and craft-related trade workers, plant and machine operators and assemblers, and elementary occupations (OR 1.98, 95% CI 1.16-3.38; *P*=.01) were significantly more likely to choose alcohol compared to the reference group, whereas students in vocational grammar school (OR 1.23, 95% CI 0.73-2.09; *P*=.43) and vocational preparation (OR 1.57, 95% CI 0.86-2.87; *P*=.14) did not differ significantly from the reference group in their likelihood of choosing alcohol. Clerical support workers were most likely to select the cannabis module (OR 3.66, 95% CI 1.33-10.03; *P*=.01), and service and sales workers were least likely to select the social competencies module (OR 0.51, 95% CI 0.30-0.88; *P*=.02) when professionals, technicians, and associate professionals were used as the reference group.

Prevention needs for each behavior were significantly related to the corresponding module choice (eg, the higher the alcohol consumption, the more likely it was that the alcohol module would be chosen; OR 1.31, 95% CI 1.21-1.43; *P*<.001).

### Use of the German Classification of Occupations to Analyze Determinants of Prevention Needs and Module Choice

The prevalence of the occupational tracks based on the German Classification of Occupations is shown in Table S8 in Multimedia Appendix 1.

Wald tests indicate that only problematic internet use (*P*=.002) and the odds of tobacco use (*P*=.002) were significantly associated with educational track after adjusting for gender and age (Table S9 in Multimedia Appendix 1). All educational tracks showed numerically higher problematic internet use than the reference category of production and construction. However, these differences did not reach significance for the tracks of natural science, geography, and computer science (regression coefficient=0.44, 95% CI −0.43 to 1.31; *P*=.32) and trading and hotel and tourism (regression coefficient=0.65, 95% CI −0.04 to 1.34; *P*=.06).

The odds of tobacco use were significantly lower in natural science, geography, and computer science (OR 0.58, 95% CI 0.34-0.97; *P*=.04); organization and accounting and media (OR 0.59, 95% CI 0.38-0.92; *P*=.02); and vocational grammar school (OR 0.52, 95% CI 0.37-0.72; *P*<.001) than in the reference category of production and construction.

Comparisons between the other educational tracks revealed that the odds of tobacco use were lower in vocational grammar school than in traffic, logistics, and security and safety (OR 0.51, 95% CI 0.30-0.89; *P*=.02); trading and hotel and tourism (OR 0.51, 95% CI 0.34-0.76; *P*<.001); health, social affairs, teaching, and education (OR 0.65, 95% CI 0.44-0.97; *P*=.03); and vocational preparation (OR 0.64, 95% CI 0.43-0.95; *P*=.03). Similarly, the odds were also lower in organization and accounting and media than in trading and hotel and tourism (OR 0.58, 95% CI 0.35-0.95; *P*=.03).

Choice of the social competencies module could not be modeled using multilevel logistic regression as the inclusion of the independent variables led to nonconvergence of the model. Instead, multiple logistic regression was used. Wald tests indicated that only the odds of choosing the social competencies (*P*<.001) and alcohol (*P*=.01) modules were significantly associated with educational track after adjusting for gender, age, and the corresponding prevention needs (Table S10 in Multimedia Appendix 1). The social competencies module was chosen significantly more often by students from the natural science, geography, and computer science tracks (OR 3.40, 95% CI 1.71-6.73; *P*<.001) and significantly less often by students from the traffic, logistics, and security and safety tracks (OR 0.32, 95% CI 0.11-0.99; *P*=.048) compared to the reference group. Comparisons between the other educational tracks revealed that all educational tracks were significantly less likely to choose the social competencies module compared to students in natural science, geography, and computer science. Students in organization, accounting and media, and vocational grammar school were significantly more likely to choose the social competencies module than students in traffic, logistics, and security and safety, as well as students in trading and hotel and tourism, whereas the other educational tracks did not differ significantly from each other in their odds of choosing the social competencies module (data not shown).

The alcohol module was selected significantly less often by students from the natural science, geography, and computer science tracks (OR 0.22, 95% CI 0.09-0.59; *P*=.002) compared to the reference group. The other educational tracks did not differ significantly from each other in their odds of choosing the alcohol module.

Although Wald tests did not reach significance, there were individual educational tracks that differed in their odds of choosing the stress, social media and gaming, and cannabis modules when compared to the reference category. Stress was chosen significantly more often by students from the health, social affairs, teaching, and education tracks (OR 2.10, 95% CI 1.11-3.98; *P*=.02), and social media and gaming was chosen significantly more often by students from the organization and accounting and media tracks (OR 2.08, 95% CI 1.16-3.72; *P*=.01). The cannabis module was chosen significantly more often by students from the traffic, logistics, and security and safety tracks (OR 4.41, 95% CI 1.54-11.13; *P*=.005). The results for the other potential determinants in the multiple regression models are consistent with those of the main analysis.

## Discussion

### Principal Findings

This study examined the prevention needs of German vocational school students and their module choices within the app-based addiction prevention intervention ready4life. Key findings included, first, that prevention needs among vocational school students were high. Almost 80% reported 2 or more risks according to yellow or red traffic light feedback. The highest prevention needs were identified for stress, social media and gaming, and alcohol. Second, in line with this, the most frequently chosen modules were stress and social media and gaming. Third, alignment of module choice with the risk feedback received was high, especially for those with 1 or 2 risks. Fourth, gender, age, educational background, and the corresponding prevention needs were associated with module choice.

### Interpretation of the Results and Comparison With Prior Work

#### Prevention Needs

Our findings regarding the prevention needs related to substance and internet use are consistent with previous epidemiological data showing a high prevalence of addictive behaviors among vocational school students [5,35,57,58], higher levels of substance use among men than among women, and high levels of substance use among students in construction tracks [5,57].

The proportions of students who received green, yellow, and red traffic light feedback, as well as the determinants of prevention needs, are congruent with those found in the Swiss evaluation study of ready4life [17]. For example, Paz Castro et al [17] also found that more than half (57.2%) of the students showed high-risk internet use, approximately a quarter (23.9%) showed high-risk tobacco use, and <10% (9.6%) showed high-risk cannabis consumption. Our study yielded approximately 12.5% more students with a high risk (red traffic light) than the study by Paz Castro et al [17] only in the case of alcohol consumption, probably due to a higher average age in our study. The high need for prevention in relation to stress is in line with previous research showing that vocational school students have high levels of job-related stress and that this is associated with substance use [50].

In line with our study, Paz Castro et al [17] found that women reported more stress and problematic internet use but less social competencies and substance use than men, problematic internet use was associated with younger age, and tobacco use was associated with older age. However, in contrast to our findings, Paz Castro et al [17] also found slightly higher rates of risky alcohol use in women than in men and higher stress as well as higher rates of risky cannabis use in older than in younger students. These differences may be explained by the different definitions of outcomes or potential determinants (eg, continuous vs categorical substance use or age) or different adjustment variables.

Comparisons of prevention needs according to educational track between these studies are difficult because different systems were used to classify occupations. However, both studies are consistent in that the areas of construction and production were among those with the highest levels of tobacco use and those with the lowest levels of problematic internet use. In both studies, cannabis consumption did not differ by educational track.

#### Module Choice

Stress and social media and gaming were the most frequently selected modules. This is congruent with the high proportion of students in our study with prevention needs in this area. The results regarding module choice were also congruent with the results of the Swiss evaluation study of ready4life [17], which had the highest choice rates for stress and social media and gaming as well.

Substance-related modules such as alcohol, tobacco, and cannabis were less frequently chosen. This is consistent with previous multibehavior interventions among adults showing that health-promoting behaviors such as physical activity and diet are preferred over substance-related modules [14,15,26,28-31] but also with interventions among vocational school students showing that stress and non–substance-related behaviors such as social media use were chosen more often than substance-related behaviors such as smoking, alcohol or cannabis use [8,17].

However, as it was intended, the traffic light feedback did affect module choice. When a module was recommended to the student based on the presence of a moderate or high risk, the choice rates became much higher. This was particularly true for those students with only 1 or 2 risks. This is consistent with previous research showing that individuals are more likely to choose modules that are recommended to them based on their risk profile [17,29]. This finding is not trivial because vocational school students usually have low motivation to change addictive behaviors [5]. This could lead students to choose life skill modules over addiction-related modules even when given feedback on their high prevention needs in terms of addictive behaviors. Furthermore, in mobile phone–based substance use interventions, those with higher severity have sometimes shown lower engagement [59]. Finally, a similar intervention found that only half of the students chose modules congruent with their risks [60] when no feedback on prevention needs was provided.

In this study, the proportion of students choosing modules congruent with their presented risk was higher (188/306, 61.4%-226/232, 97.4%). However, due to the design of the study, we cannot exclude the possibility that module choice would have been the same without module recommendation. Future experimental research should compare the effects of a choice-based system with those of a similar automated referral system on nonuse attrition and intervention effects. Previous studies have not been adequately designed to draw valid conclusions on this [26,28,30].

Choice rate was lowest for the alcohol module. Allowing users to self-select their intervention topics may leave alcohol use largely unaddressed, especially if multiple risks are present. This is in line with data from adults showing that especially at-risk alcohol users with multiple risks tend to have lower motivation to change and less interest in behavioral feedback [61]. In addition to the low motivation to change, this finding could be explained by the stigmatization of alcohol dependence [62], which may lead to a preference for less stigmatized topics. Furthermore, given the low intention to change addictive behaviors among vocational school students [5], offering the option to choose nonsubstance areas such as life skills might increase interest in participation and intervention use.

#### Potential Determinants of Module Choice

There have been some studies in adults that have analyzed potential determinants of module choice in multiple-behavior interventions [14-16]. This is the first study to statistically investigate potential determinants of module choice in vocational school students. The gender differences found in module choice are partly consistent with the prevalence differences between genders found in this study and other studies of vocational school students [17]. Furthermore, this is in line with the findings of a previous online intervention on multiple health-promoting behaviors and substance use for adults that showed higher interest of women than men in nonsubstance modules [15]. Specifically, as found in our study, in the study by Schulz et al [15], the choice of the alcohol module was related to being a man, whereas the choice of the tobacco module was not related to gender. Using the German Classification of Occupations, it is evident that differences in module choice in relation to educational track could be partly explained by the specific relevance of the modules for these tracks (eg, high stress levels in the health and education sector [63] or the influence of cannabis use on safe driving in the traffic and logistic sector). However, it is important to emphasize that associations between determinants and module choice exist independently of or in addition to the students’ prevention needs and, therefore, may also reflect how effectively at-risk groups are being reached, highlighting the issue of social equity in mobile health interventions. Many mobile interventions fail to equitably engage subpopulations, particularly those of a lower socioeconomic status [36], which can exacerbate health disparities. In this study, gender disparities in module choice were evident. For example, men were less likely to choose the stress module, whereas women were less likely to choose modules on social media and gaming, alcohol, and cannabis. Research suggests that stress is more strongly associated with substance use among men [64], making them more likely to externalize stress through substances, particularly alcohol. Failure to address stress through appropriate modules could lead to the development or continuation of these harmful coping mechanisms among men. The lower likelihood of women choosing the alcohol or cannabis modules compared to men is concerning given the increasing trends in substance use among women [65] and evidence that women are more vulnerable to the negative consequences of substance use than men [66]. This disparity in module choice may reflect perceived gender norms or social stigma.

Interestingly, students from educational tracks typically associated with lower socioeconomic status (eg, clerical support workers, service and sales workers, craft-related trade workers, and vocational preparation) were equally or even more likely to choose modules related to stress and substance use than their peers from educational tracks typically associated with higher socioeconomic status (eg, professionals, technicians, and vocational grammar school). Thus, our mobile intervention approach had a positive impact on equity in that students of a lower socioeconomic status were more likely to respond to alcohol and cannabis modules than students of a higher socioeconomic status [67]. This is a significant finding given that previous research has consistently shown that individuals of a lower socioeconomic status tend to benefit less from mobile interventions due to lower participation and intervention use [36,68,69] despite higher rates of substance use [35], clustering of addictive behaviors [5], and greater substance-related harm [70]. Some lower-status educational tracks were less likely to choose the social competencies or social media and gaming modules, such as service and sales workers or craft-related trade workers. While exposure to substance use modules may have an immediate positive impact on the health of these subgroups, not choosing social skill modules may limit students’ opportunities to develop essential skills such as communication, conflict resolution, and emotional regulation, which are valuable in both professional and personal contexts.

The fact that a higher need for prevention increased the likelihood of choosing the corresponding module is in line with the results of previous studies [8,17]. Although these studies did not statistically investigate potential determinants, higher prevention needs (indicated by yellow or red traffic light feedback) led to numerically higher choices of the corresponding modules compared to the overall average.

### Strengths

The strengths of this study include (1) the focus on vocational school students, an underserved group in mobile intervention research; (2) the use of a novel multibehavioral approach that combined life skill training with addressing multiple addictive behaviors; (3) the use of a facilitated access method that ensured a more representative sample compared to relying on online or media recruitment [40]; and (4) being the first study to statistically analyze the determinants of module choice among vocational school students.

### Limitations

The following limitations of this study should be considered when interpreting the results. First, the data may not be fully representative of German vocational school students due to selection bias (eg, additional analyses showed that men and students from nonprofessional backgrounds were less likely to participate in this study). Furthermore, not all educational tracks were represented in the sample (eg, the agriculture, forestry, or military tracks were not included). Second, data collection during the COVID-19 pandemic may have influenced participation willingness, addictive behaviors, or module selection. For example, gaming and social media use were elevated during the pandemic in adolescents [71]. Third, all assessments were based on self-report measures. Thus, there is the potential for over- or underreporting, which could have affected the accuracy of the risk and competence feedback. Fourth, this study focused on module choice rather than module completion or frequency of app use. However, completion rates are likely to differ between modules [15,31]. Fifth, we cannot exclude the possibility that the high interest in the stress module was partly triggered by the classes’ introductions, which were largely focused on stress. Finally, the number of potential determinants assessed was limited. Other variables such as students’ personal values and goals, subjective importance of health, perceptions of social norms, or migration background could also be related to their prevention needs or module choices.

### Conclusions

Our study supports the existing evidence that vocational school students have high prevention needs in relation to addictive behaviors. An important finding was that the students’ module choices were highly congruent with their identified prevention needs. This is important because intervention effects are likely to be higher when modules that are more relevant to the participant are chosen (eg, when smokers choose to work on the smoking module [60]). Our findings also highlight that incorporating self-tailoring options into mobile health interventions works well to address multiple risks and different combinations of risks for vocational school students. This is particularly important given that a high proportion of vocational school students engage in multiple addictive behaviors and may not be able to address all their issues at once. In terms of educational track, our mobile intervention approach was able to achieve a positive equity impact (ie, those in tracks typically associated with a lower socioeconomic status were more likely to choose modules related to alcohol and cannabis). The identified module preferences may inform the further development of mobile interventions to improve adherence and effectiveness within specific subgroups. To promote more gender-equitable engagement, mobile interventions need to address disparities in module selection. Future interventions for vocational school students could tailor module content or descriptions to gender to ensure that both genders engage with modules that meet their prevention needs. For example, framing stress management around work performance for men or linking substance use prevention to emotional well-being for women may increase engagement. Alternatively, embedding cross-links within modules, such as mentioning substance use in life skill modules, could promote more holistic behavior change across diverse groups. Future research should also investigate whether the choice of a particular module influences change in the behaviors addressed in other unchosen modules (eg, the effect of choosing the stress module on addictive behaviors).

## Appendix

Multimedia Appendix 1 Supplementary tables.

Multimedia Appendix 2 CONSORT-eHEALTH checklist (V 1.6.1).

Multimedia Appendix 3 STROBE (Strengthening the Reporting of Observational Studies in Epidemiology) checklist.

Multimedia Appendix 4 TIDieR (Template for Intervention Description and Replication) checklist.

## Abbreviations

CIUS: Compulsive Internet Use Scale
ICC: intraclass correlation coefficient
ISCO-8: International Standard Classification of Occupations 2008
OR: odds ratio

## Data Availability

The datasets generated or analyzed during this study are available from the corresponding author on reasonable request.
