# Supplementary material for: Prevention Needs and Target Behavior Preferences in an App-Based Addiction Prevention Program for German Vocational School Students: Cluster Randomized Controlled Trial
Source: JMIR Mhealth Uhealth. 2025 Jun 24;13:e59573. doi: 10.2196/59573 (PMC12238790; doi:10.2196/59573)
Supplement: Multimedia Appendix 1 [file mhealth_v13i1e59573_app1.doc]

### Multimedia Appendix 1

Table S1. Topic specific thresholds for the individual risk and competence profile.

|  | **Traffic light feedback ^a^** | | |
| --- | --- | --- | --- |
| **Topic and classification criteria** | **Green** | **Yellow** | **Red** |
|  |  |  |  |
| **Stress**  Perceived stress  (1 "not at all" to 5 "very strongly") | 1 or 2 | 3 | 4 or 5 |
| **Social competencies**  Feeling secure in social situations, mean (1 “very uncertain” to 5 “very certain”) | ≥ 3.5 | > 2.5 and < 3.5 | ≤ 2.5 |
| **Social media and gaming**  Problematic internet use, sum (0 to 20) | ≤ 6 | ≥ 7 and ≤ 8 | ≥ 9 |
| **Tobacco**  Tobacco smoking or nicotine product use in the last 30 days | “never” | “occasionally but not daily” | “(almost) daily“ |
| **Cannabis**  Lifetime and last six months consumption of THC-containing cannabis | “no, never” in lifetime or  “not at all” within the last six months | “once a month or less“ or “2-4 times a month“ within the last six months | “2-3 times a week“, or “4 times a week or more“ within the last six months |
| **Alcohol** | **Men** ≥ **18 years** |  |  |
| Consumption days within the past months, Number of standard drinks per drinking day, Maximum number of standard drinks per occasion,  Number of standard drinks per months | consumption days ≤ 20 and drinks per drinking day ≤ 2 and maximum number of drinks ≤ 2 | (consumption days > 20 or drinks per drinking day > 2 or maximum number of drinks > 2) and drinks per month ≤ 40 and maximum number of drinks ≤ 5 | drinks per month > 40 or maximum number of drinks > 5 |
|  | **Men aged 16 and 17 years** | | |
|  | consumption days ≤ 10 and drinks per drinking day ≤ 2 and maximum number of drinks ≤ 2 | (consumption days > 10 or drinks per drinking day > 2 or  maximum number of drinks > 2) and drinks per month ≤ 20 and maximum number of drinks ≤ 5 | drinks per month > 20 or maximum number of drinks > 5 |
|  | **Women** ≥ **18 years** |  |  |
|  | consumption days ≤ 20 and drinks drinking per day ≤ 1 and maximum number of drinks ≤ 1 | (consumption days > 20 or drinks per drinking day > 1 or  maximum number of drinks > 1) and drinks per month ≤ 20 and maximum number of drinks ≤ 4 | drinks per month > 20 or maximum number of drinks > 4 |
|  | **Women aged 16 and 17 years** | | |
|  | consumption days ≤ 10 and drinks per drinking day ≤ 1 and maximum number of drinks ≤ 1 | (consumption days > 10 or drinks per drinking day > 1 or  maximum number of drinks > 1) and drinks per month ≤ 10 and maximum number of drinks ≤ 4 | drinks per month > 10 or maximum number of drinks > 4 |

^a^ Traffic lights were based on self-reported behaviors and gave advice for potential useful modules; however, vocational students could freely select any two of the six modules.

Table S2. German Classification of Occupations.

| **Occupational areas** | **Included Occupations** |
| --- | --- |
| 1 Agriculture, forestry, animal husbandry and horticulture | Not present in the sample |
| 2 Raw material extraction, production and manufacturing | - Agricultural and industrial machinery mechanics and repairers - Bicycle and related repairers - Building and related electricians - Cabinet-makers and related workers - Cooks - Draughtspersons - Electrical mechanics and fitters - Electronics mechanics and servicers - Electronics mechanics and servicers - Food and related products machine operators - Information and communications technology installers and servicers - Motor vehicle mechanics and repairers - Paper products machine operators - Physical and engineering science technicians not elsewhere classified - Plastic products machine operators - Pre-press technicians - Precision-instrument makers and repairers - Print finishing and binding workers - Printers - Rubber products machine operators - Stationary plant and machine operators not elsewhere classified - Structural metal preparers and erectors - Toolmakers and related workers |
| 3 Construction, architecture, surveying and building technology | - Bricklayers and related workers - Carpenters and joiners - Earthmoving and related plant operators - Painters and related workers - Plumbers and pipe fitters - Roofers |
| 4 Natural science, geography and computer science | - Applications programmers - Chemical and physical science technicians - Chemical products plant and machine operators - Civil engineering technicians - Information and communications technology user support technicians |
| 5 Traffic, logistics, security and safety | - Cleaners and helpers in offices, hotels and other establishments - Heavy truck and lorry drivers - Stock clerks - Transport clerks |
| 6 Commercial services, trade in goods, distribution, hotel and tourism | - Administrative and executive secretaries - Food service counter attendants - Hotel receptionists - Shop sales assistants - Waiters |
| 7 Company organization, accounting, law and administration | - Accounting and bookkeeping clerks - Administrative and executive secretaries - Bank tellers and related clerks - Clerical support workers not elsewhere classified - General office clerks - Legal secretaries - Secretaries |
| 8 Health, social affairs, teaching and education | - Dental assistants and therapists - Domestic housekeepers, Cleaning and housekeeping supervisors in offices, hotels and other establishments - Early childhood educators - Hairdressers - Home-based personal care workers - Medical and dental prosthetic technicians - Nursing associate professionals - Social work associate professionals |
| 9 Linguistics, literature, humanities, social and economic sciences, media, art, culture and design | - Contact center information clerks |
| 0 Military | Not present in the sample |

Table S3. Classification of occupations based on ISCO-08.

| **ISCO-8 Major Groups** | **Included Occupations** |
| --- | --- |
|  |  |
| 1 Managers | Not present in the sample |
| 2 Professionals | - Applications programmers - Early childhood educators |
| 3 Technicians and associate professionals | - Administrative and executive secretaries - Chemical and physical science technicians - Civil engineering technicians - Dental assistants and therapist - Draughts persons - Information and communications technology user support technicians - Legal secretaries - Medical and dental prosthetic technicians - Nursing associate professionals - Physical and engineering science technicians - Social work associate professionals |
| 4 Clerical support workers | - Accounting and bookkeeping clerks - Bank tellers and related clerks - Clerical support workers - Contact center information clerks - General office clerks - Hotel receptionist - Secretaries - Stock clerks - Transport clerks |
| 5 Service and sales workers | - Cooks - Domestic housekeepers and housekeeping supervisors in office, hotels and other establishments - Hairdressers - Home-based personal care workers - Shop sales assistants - Waiters |
| 6 Skilled agricultural, forestry and fishery workers | Not present in the sample |
| 7 Craft-related trades workers | - Agricultural and industrial machinery mechanics and repairers - Bicycle and related repairers - Bricklayers and related workers - Building and related electricians - Cabinet-makers and related workers - Carpenters and joiners - Electrical mechanics and fitters - Electronics mechanics and servicers - Information and communications technology installers and servicers - Motor vehicle mechanics and repairers - Painters and related workers - Plumber and pipe fitters - Pre-press technicians - Precision-instrument makers and repairers - Print finishing and binding workers - Printers - Roofers - Structural metal preparers and erectors - Toolmakers and related workers |
| 8 Plant and Machine Operators and Assemblers | - Chemical products plant and machine operators - Earthmoving and related plant operators - Food and related products machine operators - Heavy truck and lorry drivers - Plastic products machine operators - Paper products machine operators - Rubber products machine operators - Plastic products machine operators - Stationary plant and machine operators |
| 9 Elementary occupations | - Cleaners and helpers in office, hotels and other establishments |
| 0 Armed forces occupations | Not present in the sample |

Table S4. Baseline description of study participants.

|  | **Total**  **(n=2568)** | **Control group**  **(n=1282)** | **Intervention group**  **(n=1286)** | **Intervention group with module choice (n=1236)** |
| --- | --- | --- | --- | --- |
|  |  |  |  |  |
| **Gender, n (%)** |  |  |  |  |
| Men | 1398 (54.4%) | 694 (54.1) | 704 (54.7%) | 671 (54.3%) |
| Women | 1170 (45.6%) | 588 (45.9%) | 582 (45.3%) | 565 (45.7%) |
| **Age, M (SD) ^a^** | 19.68 (3.65) | 19.85 (3.73) | 19.51 (3.56) | 19.53 (3.58) |
| **Perceived stress from 1 to 5, M (SD)** | 3.29 (1.20) | 3.27 (1.20) | 3.30 (1.19) | 3.31 (1.19) |
| **Social Competencies from 8 to 40, M (SD)** | 29.50 (4.69) | 29.48 (4.70) | 29.53 (4.67) | 29.54 (4.68) |
| **Problematic Internet use from 0 to 20, M (SD)** | 9.14 (4.18) | 9.10 (4.19) | 9.19 (4.17) | 9.17 (4.15) |
| **Number of alcoholic standard drinks per day, Mdn (IQR)** | 0.20 (0.00-1.00) | 0.20 (0.00-0.87) | 0.23 (0.00-1.00) | 0.23 (0.00-1.00) |
| **Tobacco use last month, n (%) ^b, c^** | 814 (31.7%) | 392 (30.6%) | 422 (32.8%) | 406 (32.9%) |
| **Cannabis use last month, n (%) ^d^** | 464 (18.1%) | 227 (17.7%) | 237 (18.4%) | 225 (18.2%) |
| **Federal state, n (%)** |  |  |  |  |
| Baden-Wuerttemberg | 198 (7.7%) | 111 (8.7%) | 87 (6.8%) | 87 (7%) |
| Mecklenburg-Western Pomerania | 55 (2.1%) | 15 (1.2%) | 40 (3.1%) | 40 (3.2%) |
| Lower Saxony | 646 (25.2%) | 374 (29.2%) | 272 (21.2%) | 263 (21.3%) |
| North-Rhine-Westphalia | 363 (14.1%) | 146 (11.4%) | 217 (16.9%) | 213 (17.2%) |
| Schleswig-Holstein | 1306 (50.9%) | 636 (49.6%) | 670 (52.1%) | 633 (51.2%) |
| **Educational track, n (%) ^e,f^** |  |  |  |  |
| Vocational training | 1648 (64.2%) | 857 (66.9%) | 791 (61.5%) | 767 (62.1%) |
| Professionals | 12 (0.5%) | 6 (0.5%) | 6 (0.5%) | 6 (0.5%) |
| Technicians and associate professionals | 511 (19.9%) | 282 (22.0%) | 229 (17.8%) | 222 (18%) |
| Clerical support workers | 287 (11.2%) | 156 (12.2%) | 131 (10.2%) | 127 (10.3%) |
| Service and sales workers | 286 (11.1%) | 137 (10.7%) | 149 (11.6%) | 148 (12%) |
| Craft-related trades workers | 422 (16.4%) | 216 (16.9%) | 206 (16.0%) | 199 (16.1%) |
| Plant and Machine Operators  and Assemblers | 66 (2.6%) | 50 (3.9%) | 16 (1.2%) | 16 (1.3%) |
| Elementary occupations | 2 (0.1%) | 2 (0.2%) | 0 (0.0%) | 0 (0%) |
| Mixed occupations | 62 (2.4%) | 8 (0.6%) | 54 (4.2%) | 49 (4.0%) |
| Vocational grammar school ^g^ | 582 (22.7%) | 287 (22.4%) | 295 (22.9%) | 282 (22.8%) |
| Vocational preparation ^h^ | 282 (11%) | 118 (9.2%) | 164 (12.8%) | 151 (12.2%) |
| **Year of education, n (%)** **^e,i^** |  |  |  |  |
| First year | 980 (38.2%) | 448 (35.0%) | 532 (41.4%) | 507 (41%) |
| Second year | 805 (31.4%) | 428 (33.4%) | 377 (29.3%) | 366 (29.6%) |
| Third year | 214 (8.3%) | 95 (7.4%) | 119 (9.3%) | 116 (9.4%) |

M, Mean; SD, Standard Deviation; Mdn, Median; IQR, Inter-Quartile-Range.

^a^ Information is missing for 1/2568 (0.04%).

^b^ Information is missing for 1/2568 (0.04%).

^c^ At least one cigarette smoked in the last month.

^d^ At least one cannabis consumption day in the last month.

^e^ Percentages do not add up to 100 due to missing information.

^f^ Information is missing for 14/2568 (0.6%) participants and 42/2568 (1.6%) students came from classes including different educational tracks.

^g^ In Germany most vocational schools also offer participation in vocational grammar school classes (typically grades 11 to 13) to prepare students for general university entrance certification.

^h^ These include vocational preparation classes as well as 1- or 2-year basic training with intermediate secondary school-leaving certificate (without training qualification).

^i^ Information is missing for 299/2568 (11.6%) students and 270/2568 (10.5%) students came from classes with different years of education.

Table S5. Association of gender, age and educational track with continuous addictive behaviors, perceived stress and social competencies. ^a-c^

| **Potential determinants** | **Perceived stress** | **Social Competencies** | **Problematic Internet use** | **Number of alcoholic standard drinks per day** | **Tobacco use last month ^f^**  **(yes vs. no)** | **Cannabis use last month ^g^**  **(yes vs. no)** |
| --- | --- | --- | --- | --- | --- | --- |
|  | **Coef.**  **(95% CI), *P*** | **Coef.**  **(95% CI), *P*** | **Coef.**  **(95% CI), *P*** | **Coef.**  **(95% CI), *P*** | **OR**  **(95% CI), *P*** | **OR**  **(95% CI), *P*** |
|  | **ICC = 8.2%** | **ICC = 5.4%** | **ICC = 5.9%** | **ICC = 13.5%** | **ICC = 9.0%** | **ICC = 2.9%** |
|  |  |  |  |  |  |  |
| **Gender (Reference: Men)** | | | | | | |
| Women | **0.70 (0.60 to 0.80), *P*<.001** | **-2.27 (-2.67 to -1.88), *P*<.001** | **0.74 (0.39 to 1.09), *P*<.001** | **-0.40 (-0.55 to -0.24), *P*<.001** | **0.79 (0.65 to 0.97), *P*=.02** | **0.60 (0.48 to 0.75), *P*<.001** |
| **Age** | 0.01 (-0.001 to 0.03), *P*=.06 | 0.05 (-0.005 to 0.10), *P*=.07 | **-0.10 (-0.15 to -0.06), *P*<.001** | 0.01 (-0.004 to 0.03), *P*=.12 | **1.04 (1.01 to 1.06), *P*=.006** | 1.02 (0.99 to 1.05), *P*=.18 |
| **Educational track (Reference: Professionals / Technicians and associate professionals)** | | | | | | |
| Clerical support workers | -0.02 (-0.22 to 0.18), *P*=.87 | **1.10 (0.36 to 1.84), *P*=.004** | 0.50 (-0.17 to 1.17), *P*=.15 | 0.13 (-0.23 to 0.49), *P*=.49 | 1.004 (0.67 to 1.50), *P*=.98 | 1.11 (0.73 to 1.67), *P*=.63 |
| Service and sales workers | 0.05 (-0.14 to 0.25), *P*=.59 | **0.79 (0.05 to 1.52),**  ***P*=.04** | -0.32 (-0.98 to 0.35), *P*=.35 | 0.30 (-0.06 to 0.65), *P*=.10 | **1.61 (1.10 to 2.37), *P*=.02** | 1.03 (0.68 to 1.56), *P*=.89 |
| Craft-related trades workers / Plant and Machine Operators and Assemblers / Elementary occupations | -0.03 (-0.21 to 0.14), *P*=.70 | 0.41 (-0.24 to 1.06), *P*=.21 | **-0.76 (-1.35 to -0.17), *P*=.01** | 0.15 (-0.16 to 0.46), *P*=.34 | 1.41 (1.0003 to 1.99), *P*=.05 | 1.28 (0.90 to 1.81), *P*=.17 |
| Vocational grammar school ^d^ | 0.14 (-0.02 to 0.31), *P*=.10 | **0.89 (0.27 to 1.52), *P*=.005** | 0.19 (-0.38 to 0.75), *P*=.56 | 0.07 (-0.23 to 0.37), *P*=.64 | 0.74 (0.52 to 1.05), *P*=.09 | 1.15 (0.81 to 1.63), *P*=.44 |
| Vocational preparation ^e^ | -0.05 (-0.26 to 0.15), *P*=.61 | **1.02 (0.26 to 1.79), *P*=.009** | 0.24 (-0.45 to 0.92), *P*=.50 | 0.27 (-0.09 to 0.63), *P*=.14 | 1.16 (0.77 to 1.75), *P*=.48 | 1.34 (0.88 to 2.03), *P*=.17 |

Coef., Regression coefficient; OR, Odds ratio; CI, confidence interval.

^a^ Displayed are results from six separate multiple multilevel regression analyses with random intercept on class level: Treating tobacco and cannabis consumption as continuous led to non-convergence when a random intercept on class-level was modelled. Hence, we reported on binary outcome variables of tobacco and cannabis consumption to ensure model convergence assuming a random-intercept on class level. Stress, social competencies and problematic internet use were modelled as linear and number of alcoholic standard drinks as negative-binomial.

^b^ Analyses were based on 2449 students due to missing values, except for tobacco use, which analysis was based on 2448 students.

^c^ Significant comparisons (*P*<.05) are displayed as bold.

^d^ These include classes that receive General university entrance certification (without training qualification).

^e^ These include vocational preparation classes as well as 1- or 2-year basic training with Intermediate secondary school-leaving certificate (without training qualification).

^f^ At least one cigarette smoked in the last month.

^g^ At least one cannabis consumption day in the last month.

Table S6. Module choice by number of risks according to traffic light feedback (n=1236). a

|  | **Module choice, n (%)** | | | | | |
| --- | --- | --- | --- | --- | --- | --- |
| **Number of risks based on** | **Stress** | **Social competencies** | **Social media and gaming** | **Alcohol** | **Tobacco** | **Cannabis** |
|  |  |  |  |  |  |  |
| **Yellow** /r**ed traffic light feedback** |  |  |  |  |  |  |
| One or two  including the respective behavior ^b^ | 193/200 (96.5%) | 40/41  (97.6%) | 193/206 (93.7%) | 124/175 (70.9%) | 35/41  (85.4%) | 13/19  (68.4%) |
| More than two  including the respective behavior ^b^ | 729/907 (80.4%) | 188/368 (51.1%) | 542/884 (61.3%) | 345/822 (42.0%) | 228/554 (41.2%) | 117/323 (36.2%) |
| **Red traffic light feedback only** |  |  |  |  |  |  |
| One or two  including the respective behavior ^b^ | 302/318 (95.0%) | 7/7  (100.0%) | 316/389 (81.2%) | 180/292 (61.6%) | 71/98  (72.5%) | 14/18  (77.8%) |
| More than two  including the respective behavior ^b^ | 543/619 (87.7%) | 22/27  (81.5%) | 452/689 (65.6%) | 275/590 (46.6%) | 191/329 (58.1%) | 68/94  (72.3%) |

^a^ Traffic lights were based on self-reported behaviors and gave advice for potential useful modules; however, vocational students could freely select any two of the six modules.

^b^ n (%) of students that chose a specific module, e.g., stress, by different numbers of risks, when the specific behavior was one of the shown risks, e.g., n=200 students of the sample had 1 or 2 risks (yellow/red traffic lights) including stress, of them 96.5% (193/200) chooses the stress module to be coached in.

Table S7. Association of gender, age, educational track and prevention needs with module choice. a-c

|  | **Module choice (0 = not chosen vs. 1 = chosen)** | | | | | |
| --- | --- | --- | --- | --- | --- | --- |
| **Potential determinants** | **Stress** | **Social competencies** | **Social media**  **and gaming** | **Alcohol** | **Tobacco** | **Cannabis** |
|  | **OR**  **(95% CI), *P*** | **OR**  **(95% CI), *P*** | **OR**  **(95% CI), *P*** | **OR**  **(95% CI), *P*** | **OR**  **(95% CI), *P*** | **OR**  **(95% CI), *P*** |
|  | **ICC = 11.0%** | **ICC = 9.1%** | **ICC = 2.1%** | **ICC = 10.8%** | **ICC = 9.6%** | **ICC = 5.7%** |
| **Gender (Reference: Men)** | | | | | | |
| Women | **2.38 (1.69 - 3.33), *P*<.001** | 1.13 (0.83 - 1.54), *P*=.44 | **0.52 (0.40 - 0.69), *P*<.001** | **0.50 (0.37 - 0.67), *P*<.001** | 1.01 (0.70 - 1.46), *P*=.97 | **0.37 (0.21 - 0.63), *P*<.001** |
| **Age** | 1.02 (0.97 - 1.07), *P*=.40 | 1.02 (0.98 - 1.07), *P*=.30 | 0.96 (0.92 - 1.00), *P*=.07 | 0.97 (0.93 - 1.02), *P*=0.25 | 1.02 (0.96 - 1.07), *P*=.57 | **0.81 (0.74 - 0.90), *P*<.001** |
| **Educational track (Reference: Professionals / Technicians and associate professionals)** | | | | | | |
| Clerical support workers | 0.78 (0.41 - 1.50), *P*=.46 | 0.59 (0.34 - 1.04), *P*=.07 | 1.19 (0.70 - 2.00), *P*=.53 | **1.85 (1.02 - 3.38), *P*=.04** | 1.10 (0.51 - 2.33), *P*=.81 | **3.66 (1.33 - 10.03), *P*=.01** |
| Service and sales workers | 0.84 (0.45 - 1.57), *P*=.58 | **0.51 (0.30 - 0.88), *P*=.02** | 0.71 (0.43 - 1.16), *P*=.17 | **1.77 (1.00 - 3.15), *P*=.049** | 1.55 (0.79 - 3.02), *P*=.20 | 1.86 (0.64 - 5.43), *P*=.26 |
| Craft-related trades workers / Plant and Machine Operators and Assemblers / Elementary occupations | 0.66 (0.37 - 1.16), *P=.*15 | 0.64 (0.39 - 1.03), *P=.*07 | **0.61 (0.39 - 0.97), *P*=.04** | **1.98 (1.16 - 3.38), *P=.*01** | 1.72 (0.92 - 3.21), *P=.*09 | 1.55 (0.59 - 4.08), *P=.*37 |
| Vocational grammar school ^d^ | 1.04 (0.59 - 1.84), *P=.*88 | 0.87 (0.55 - 1.36), *P=.*53) | 0.72 (0.46 - 1.11), *P*=0.13 | 1.23 (0.73 - 2.09), *P=.*43 | 1.06 (0.56 - 1.99), *P=.*87 | 2.04 (0.82 - 5.09), *P=.*13 |
| Vocational preparation ^e^ | 0.66 (0.35 - 1.26), *P=.*21 | 0.62 (0.36 - 1.07), *P=.*09 | 0.70 (0.42 - 1.17), 0.17 | 1.57 (0.86 - 2.87), *P=.*14 | 1.49 (0.75 - 2.97), *P=.*26 | 1.30 (0.45 - 3.77), *P=.*63 |
| **Prevention need for the respective behavior** | **2.94 (2.52 - 3.43), *P*<.001** | **0.83 (0.80 - 0.86), *P*<.001** | **1.22 (1.18 - 1.26), *P*<.001** | **1.31 (1.21 - 1.43), *P*<.001** | **1.22 (1.18 - 1.26), *P*<.001** | **1.26 (1.20 - 1.31), *P*<.001** |

^a^ Displayed are results from separate multiple multilevel logistic regression analyses with random intercept on class level.

^b^ Analyses were based on 1153 students due to missing values.

^c^ ORs in bold indicate significant associations (*P*<.05).

^d^ These include classes that receive General university entrance certification (without training qualification).

^e^ These include vocational preparation classes as well as 1- or 2-year basic training with Intermediate secondary school-leaving certificate (without training qualification).

Table S8. Baseline description of the sample according to educational track when using German Classification of Occupation.

|  | **Total**  **(n= 2568)** | **Control group**  **(n=1282)** | **Intervention group (n=1286)** | **Intervention group with module choice (n=1236)** |
| --- | --- | --- | --- | --- |
|  |  |  |  |  |
| **Educational track, n (%) ^a,b^** |  |  |  |  |
| Vocational training | 1648 (64.2%) | 857 (66.9%) | 791 (61.5%) | 767 (62.1%) |
| Raw material extraction, production and manufacturing | 459 (17.9%) | 262 (20.4%) | 197 (15.3%) | 191 (15.5%) |
| Construction, architecture, surveying and building  technology | 63 (2.5%) | 24 (1.9%) | 39 (3.0%) | 38 (3.1%) |
| Natural science, geography and computer science | 131 (5.1%) | 77 (6.0%) | 54 (4.2%) | 51 (4.1%) |
| Traffic, logistics, security and safety | 125 (4.9%) | 80 (6.2%) | 45 (3.5%) | 43 (3.5%) |
| Commercial services, trade in goods, distribution, hotel and  tourism | 264 (10.3%) | 117 (9.1%) | 147 (11.4%) | 146 (11.8%) |
| Company organization, accounting, law and administration | 202 (7.9%) | 117 (9.1%) | 85 (6.6%) | 83 (6.7%) |
| Health, social affairs, teaching and education | 335 (13.1%) | 170 (13.3%) | 165 (12.8%) | 161 (13.0%) |
| Linguistics, literature, humanities, social and economic  sciences, media, art, culture and design | 7 (0.3%) | 2 (0.2%) | 5 (0.4%) | 5 (0.4%) |
| Mixed occupations | 62 (2.4%) | 8 (0.6%) | 54 (4.2%) | 49 (4.0%) |
| Vocational grammar school ^c^ | 582 (22.7%) | 287 (22.4%) | 295 (22.9%) | 282 (22.8%) |
| Vocational preparation ^d^ | 282 (11.0%) | 118 (9.2%) | 164 (12.8%) | 151 (12.2%) |

^a^ Percentages do not add up to 100 due to missing information.

^b^ Information is missing for 14/2568 (0.6%) participants and 42/2568 (1.6%) students came from classes including different educational tracks.

^c^ In Germany most vocational schools also offer participation in vocational grammar school classes (typically grades 11 to 13) to prepare students for general university entrance certification.

^d^ These include vocational preparation classes as well as 1- or 2-year basic training with intermediate secondary school-leaving certificate (without training qualification).

Table S9. Association of gender, age and educational track with continuous addictive behaviors, perceived stress and social competencies when using German Classification of Occupations. a-c

| **Potential determinants** | **Perceived stress** | **Social Competencies** | **Problematic Internet use** | **Number of alcoholic standard drinks per day** | **Tobacco use last month ^i^**  **(yes vs. no)** | **Cannabis use in the last month ^j^**  **(yes vs. no)** |
| --- | --- | --- | --- | --- | --- | --- |
|  | **Coef.**  **(95% CI), *P*** | **Coef.**  **(95% CI), *P*** | **Coef.**  **(95% CI), *P*** | **Coef.**  **(95% CI), *P*** | **OR**  **(95% CI), *P*** | **OR**  **(95% CI), *P*** |
|  | **ICC = 8.2%** | **ICC = 5.4%** | **ICC = 5.9%** | **ICC = 13.5%** | **ICC = 9.0%** | **ICC = 2.9%** |
| **Gender (Reference: Men)** | | | | | | |
| Women | **0.72 (0.62 to 0.82), *P<.*001** | **-2.39 (-2.79 to -1.99), *P<.*001** | **0.67 (0.31 to 1.03), *P<.*001** | **-0.41 (-0.57 to -0.26), *P<.*001** | **0.79 (0.65 to 0.98), *P=.*028** | **0.60 (0.47 to 0.76), *P<.*001** |
| **Age** | 0.01 (-0.001 to 0.03), *P=.*08 | 0.04 (-0.01 to 0.10), *P=.*10 | **-0.11 (-0.16 to -0.06), *P<.*001** | 0.01 (-0.003 to 0.03), *P=.*10 | **1.04 (1.01 to 1.06), *P=.*007** | 1.02 (0.99 to 1.05), *P=.*21 |
| **Educational track (Reference: Production / Construction ^d^)** | | | | | | |
| Natural science, geography and computer science | 0.16 (-0.10 to 0.42), *P=.*23 | -0.67 (-1.64 to 0.30), *P=.*17 | 0.44 (-0.43 to 1.31), *P=.*32 | -0.39 (-0.85 to 0.08), *P=.*11 | **0.58 (0.34 to 0.97), *P=.*04** | 0.77 (0.46 to 1.30), *P=.*33 |
| Traffic, logistics, security and safety | 0.10 (-0.18 to 0.37), *P=.*49 | 0.22 (-0.80 to 1.23), *P=.*67 | **1.45 (0.54 to 2.35), *P=.*002** | -0.002 (-0.49 to 0.48), *P=.*99 | 1.005 (0.59 to 1.71), *P=.*99 | 1.03 (0.62 to 1.71), *P=.*92 |
| Trading, hotel and tourism ^e^ | 0.04 (-0.17 to 0.24), *P=.*71 | 0.47 (-0.30 to 1.24), *P=.*24 | 0.65 ( -0.04 to 1.34), *P=.*06 | 0.09 (-0.27 to 0.45), *P=.*63 | 1.02 (0.69 to 1.52), *P=.*92 | 0.79 (0.52 to 1.21), *P=.*28 |
| Organization, accounting / Media ^f^ | -0.06 (-0.28 to 0.16), *P=.*62 | 0.63 (-0.20 to 1.45), *P=.*14 | **0.997 (0.26 to 1.73), *P=.*008** | -0.22 (-0.63 to 0.18), *P=.*28 | **0.59 (0.38 to 0.92), *P=.*02** | 0.66 (0.41 to 1.06), *P=.*09 |
| Health, social affairs, teaching and education | -0.02 (-0.22 to 0.18), *P=.*85 | 0.13 (-0.62 to 0.88), *P=.*73 | **1.17 (0.51 to 1.84), *P<.*001** | 0.06 (-0.29 to 0.41), *P=.*74 | 0.79 (0.53 to 1.18), *P=.*25 | 0.89 (0.59 to 1.33), *P=.*57 |
| Vocational grammar school ^g^ | 0.16 (-0.01 to 0.33), *P=.*06 | 0.57 (-0.05 to 1.20), *P=.*07 | **1.02 (0.46 to 1.58), *P<.*001** | -0.09 (-0.38 to 0.20), *P=.*56 | **0.52 (0.37 to 0.72), *P<.*001** | 0.90 (0.65 to 1.26), *P=.*55 |
| Vocational preparation ^h^ | -0.04 (-0.24 to 0.17), *P=.*72 | 0.70 (-0.06 to 1.46), *P=.*07 | **1.07 (0.38 to 1.75), *P=.*002** | 0.11 (-0.24 to 0.46), *P*=0.53 | 0.81 (0.54 to 1.21), *P=.*30 | 1.05 (0.70 to 1.57), *P=.*81 |

Coef., Regression coefficient; OR, Odds ratio; CI, confidence interval.

^a^ Displayed are results from six separate multilevel regression analyses with random intercept on class level: stress, social competencies and problematic internet use were modelled as linear, tobacco and cannabis consumption as binary and number of alcoholic standard drinks as negative-binomial.

^b^ Analyses were based on 2449 students due to missing values, except for tobacco use, which analysis was based on 2448 students.

^c^ Significant comparisons are displayed as bold.

^d^ Raw material extraction, production and manufacturing / Construction, architecture, surveying and building technology.

^e^ Commercial services, trade in goods, distribution, hotel and tourism.

^f^ Company organization, accounting, law and administration / Linguistics, literature, humanities, social and economic sciences, media, art, culture and design.

^g^ These include classes that receive General university entrance certification (without training qualification).

^h^ These include vocational preparation classes as well as 1- or 2-year basic training with Intermediate secondary school-leaving certificate (without training qualification).

^i^ At least one cigarette smoked in the last month.

^j^ At least one cannabis consumption day in the last month.

Table S10. Association of gender, age, educational track and prevention needs with module choice when using German Classification of Occupations. a-c

|  | **Module choice (0 = not chosen vs. 1 = chosen)** | | | | | |
| --- | --- | --- | --- | --- | --- | --- |
| **Potential determinants** | **Stress** | **Social competencies** | **Social media and gaming** | **Alcohol** | **Tobacco** | **Cannabis** |
|  | **OR**  **(95% CI), *P*** | **OR**  **(95% CI), *P*** | **OR**  **(95% CI), *P*** | **OR**  **(95% CI), *P*** | **OR**  **(95% CI), *P*** | **OR**  **(95% CI), *P*** |
|  | **ICC = 11.0%** | **ICC = 9.1%** | **ICC = 2.1%** | **ICC = 10.8%** | **ICC = 9.6%** | **ICC = 5.7%** |
| **Gender (Reference: Men)** | | | | | | |
| Women | **2.15 (1.52 - 3.03), *P<.*001** | 1.31 (0.95 - 1.80), *P=.*10 | **0.51 (0.39 - 0.68), *P<.*001** | **0.48 (0.35 - 0.65), *P<.*001** | 0.95 (0.65 - 1.39), *P=.*79 | **0.39 (0.22 - 0.68), *P<.*001** |
| **Age** | 1.02 (0.97 - 1.07), *P=.*38 | 1.03 (0.99 - 1.08), *P=.*14 | 0.96 (0.93 - 1.00), *P=.*07 | 0.97 (0.92 - 1.01), *P=.*17 | 1.01 (0.96 - 1.07), *P=.*67 | **0.81 (0.73 - 0.89), *P<.*001** |
| **Educational track (Reference: Production / Construction ^d^)** | | | | | | |
| Natural science, geography and computer science | 0.96 (0.42 - 2.19), *P=.*92 | **3.40 (1.71 - 6.73), *P<.*001** | 1.23 (0.61 - 2.49), *P=.*56 | **0.22 (0.09 - 0.59), *P=.*002** | 0.52 (0.18 - 1.54), *P=.*24 | 0.64 (0.17 - 2.44), *P=.*51 |
| Traffic, logistics, security and safety | 0.55 (0.22 - 1.36), *P=.*20 | **0.32 (0.11 - 0.99), *P=.*048** | 1.46 (0.69 - 3.12), *P=.*33 | 1.40 (0.65 - 3.01), *P=.*39 | 0.92 (0.30 - 2.80), *P=.*88 | **4.14 (1.54 - 11.13), *P=.*005** |
| Trading, hotel and tourism ^e^ | 1.17 (0.65 - 2.12), *P=.*60 | 0.60 (0.34 - 1.05), *P=.*08 | 1.21 (0.74 - 1.96), *P=.*45 | 1.15 (0.69 - 1.90), *P=.*60 | 1.15 (0.60 - 2.22), *P=.*67 | 1.16 (0.47 - 2.89), *P=.*75 |
| Organization, accounting / Media ^f^ | 1.29 (0.65 - 2.58), *P=.*46 | 1.22 (0.67 - 2.23), *P=.*51 | **2.08 (1.16 - 3.72), *P=.*01** | 0.68 (0.36 - 1.30), *P=.*25 | 0.76 (0.33 - 1.77), *P=.*53 | 1.19 (0.39 - 3.64), *P=.*76 |
| Health, social affairs, teaching and education | **2.10 (1.11 - 3.98), *P=.*02** | 0.82 (0.49 - 1.37), *P=.*45 | 1.62 (0.98 - 2.67), *P=.*06 | 0.63 (0.36 - 1.11), *P=.*11 | 0.81 (0.40 - 1.62), *P=.*55 | 0.63 (0.20 - 1.96), *P=.*43 |
| Vocational grammar school ^g^ | 1.51 (0.91 - 2.50), *P=.*11 | 1.18 (0.75 - 1.83), *P=.*47 | 1.14 (0.76 - 1.72), *P=.*53 | 0.65 (0.42 - 1.02), *P=.*06 | 0.72 (0.40 - 1.30), *P=.*27 | 1.27 (0.63 - 2.55), *P=.*50 |
| Vocational preparation ^h^ | 0.99 (0.55 - 1.78), *P=.*98 | 0.83 (0.49 - 1.43), *P=.*51 | 1.12 (0.68 - 1.83), *P=.*66 | 0.83 (0.49 - 1.40), *P=.*49 | 1.02 (0.52 - 1.98), *P=.*96 | 0.79 (0.32 - 1.90), *P=.*59 |
| **Prevention need for the respective behavior** | **2.97 (2.54 - 3.46), *P<.*001** | **0.83 (0.80 - 0.86), *P<.*001** | **1.22 (1.17 - 1.26), *P<.*001** | **1.32 (1.21 - 1.43), *P<.*001** | **1.22 (1.18 - 1.26), *P<.*001** | **1.26 (1.20 - 1.31), *P<.*001** |

^a^ Displayed are results from six separate multiple multilevel logistic regression analyses with random intercept on class level, except for social competencies which was modelled without random intercept on class level due to model non-convergence when including all independent variables.

^b^ Analyses were based on 1153 students due to missing values.

^c^ ORs in bold indicate significant associations (*P<.*05).

^d^ Raw material extraction, production and manufacturing / Construction, architecture, surveying and building technology.

^e^ Commercial services, trade in goods, distribution, hotel and tourism.

^f^ Company organization, accounting, law and administration / Linguistics, literature, humanities, social and economic sciences, media, art, culture and design.

^g^ These include classes that receive General university entrance certification (without training qualification).

^h^ These include vocational preparation classes as well as 1- or 2-year basic training with Intermediate secondary school-leaving certificate (without training qualification).
